# Differential trends of admissions in accident and emergency departments during the COVID-19 pandemic in Germany

**DOI:** 10.1186/s12873-021-00436-0

**Published:** 2021-04-06

**Authors:** Philipp Jaehn, Christine Holmberg, Greta Uhlenbrock, Andreas Pohl, Thomas Finkenzeller, Michael T. Pawlik, Ivo Quack, Antonio Ernstberger, Felix Rockmann, Andreas G. Schreyer

**Affiliations:** 1 Institute of Social Medicine and Epidemiology, Brandenburg Medical School Theodor Fontane, Brandenburg, Germany; 2 Faculty of Health Sciences Brandenburg, Brandenburg Medical School Theodor Fontane, Brandenburg, Germany; 3 Department of Emergency Medicine, Kliniken Nordoberpfalz, Weiden, Germany; 4 Department of Radiology, Kliniken Nordoberpfalz, Weiden, Germany; 5 Department of Anesthesiology, Caritaskrankenhaus St. Josef, Regensburg, Germany; 6 Department of Emergency Medicine, Klinikum Konstanz, Konstanz, Germany; 7 Department of Trauma Surgery, Center for Musculoskeletal Surgery, Klinikum Osnabrueck, Osnabrueck, Germany; 8 Department of Emergency Medicine, Krankenhaus Barmherzige Brüder, Regensburg, Germany; 9 Institute of Diagnostic and Interventional Radiology, Brandenburg Medical School Theodor Fontane, Brandenburg, Germany

**Keywords:** Accident and emergency, Admission rate, COVID-19, Internal medicine, Surgery, Mental health, Trauma

## Abstract

**Background:**

Recent studies have shown a decrease of admissions to accident and emergency (A&E) departments after the local outbreaks of COVID-19. However, differential trends of admission counts, for example according to diagnosis, are less well understood. This information is crucial to inform targeted intervention. Therefore, we aimed to compare admission counts in German A&E departments before and after 12th march in 2020 with 2019 according to demographic factors and diagnosis groups.

**Methods:**

Routine data of all admissions between 02.12.2019–30.06.2020 and 01.12.2018–30.06.2019 was available from six hospitals in five cities from north-western, eastern, south-eastern, and south-western Germany. We defined 10 diagnosis groups using ICD-10 codes: mental disorders due to use of alcohol (MDA), acute myocardial infarction (AMI), stroke or transient ischemic attack (TIA), heart failure, pneumonia, chronic obstructive pulmonary disease (COPD), cholelithiasis or cholecystitis, back pain, fractures of the forearm, and fractures of the femur. We calculated rate ratios comparing different periods in 12.03.2020–30.06.2020 with 12.03.2019–30.06.2019.

**Results:**

Forty-one thousand three hundred fifty-three cases were admitted between 12.03.2020–30.06.2020 and 51,030 cases between 12.03.2019–30.06.2019. Admission counts prior to 12.03. were equal in 2020 and 2019. In the period after 12.03., the decrease of admissions in 2020 compared to 2019 was largest between 26.03. and 08.04. (− 30%, 95% CI − 33% to − 27%). When analysing the entire period 12.03.-30.06., the decrease of admissions was heterogeneous among hospitals, and larger among people aged 0–17 years compared to older age groups. In the first 8 weeks after 12.03., admission counts of all diagnoses except femur fractures and pneumonia declined. Admissions with pneumonia increased in this early period. Between 07.05. and 30.6.2020, we noted that admissions with AMI (+ 13%, 95% CI − 3% to + 32%) and cholelithiasis or cholecystitis (+ 20%, 95% CI + 1% to + 44%) were higher than in 2019.

**Conclusions:**

Our results suggest differential trends of admission counts according to age, location, and diagnosis. An initial decrease of admissions with MDA, AMI, stroke or TIA, heart failure, COPD, cholelithiasis or cholecystitis, and back pain imply delays of emergency care in Germany. Finally, our study suggests a delayed increase of admissions with AMI and cholelithiasis or cholecystitis.

## Background

On September 14th 2020, the World Health Organization has registered 28,584,158 confirmed cases of coronavirus disease 2019 (COVID-19), and 916,955 deaths due to COVID-19 worldwide [1]. The new coronavirus (SARS-CoV-2) that causes COVID-19 continues to challenge public health systems across the globe. On March 12th 2020, the German federal government announced that hospitals were to close down their planned and non-urgent day-to-day routine in order to prepare for the rising numbers of COVID-19-cases at that time [2]. On 22nd of March, Germany went into a national lockdown and introduced comprehensive measures of social distancing [3].

It has been reported from many countries that admissions to accident and emergency (A&E) departments declined during the beginning of the outbreak of COVID-19 in their respective countries [4, 5]. In its latest situation report, the German public health institute (Robert Koch-Institute) describes a decline of up to 40% in daily admissions at national A&E departments from mid-March onwards [6]. Similarly, a recently published study describes a significant decrease in admissions at A&E departments, with a maximum decrease of 38% in the week of the highest number of daily incident COVID-19 cases in Germany [7]. There are three potential reasons for the decline of admissions at A&E departments: incidence of conditions that need emergency treatment could have changed, the public may have avoided hospital visits, or health care systems changed the organization of A&E departments during lockdown [8].

Some studies on incidence trends during the outbreak of COVID-19 have been published so far. For example in Germany, there is some evidence that incidence of acute pharyngitis, bronchitis and pneumonia declined during lockdown [9]. Furthermore, the incidence of traumatic injuries has decreased significantly during enforced social distancing in Germany and northern Italy [10, 11]. In contrast, studies from Germany, the US, and Australia describe a rising prevalence of depression, mental distress, and alcohol use, which is hypothesized to be due to the enforcement of drastic changes to daily living due to COVID-19 [12–16].

Findings from several studies have suggested an increase of mortality rates during the first wave of the pandemic. With regards to observational studies on all-cause excess mortality, a recent study showed an excess of 8071 deaths in Germany during the first 2 months of the pandemic [17]. In a city in northern Italy that was severely affected by COVID-19, all-cause mortality rose considerably during the outbreak in March [18]. Furthermore, Wu and colleagues describe an excess of acute cardiovascular mortality of 2085 (+ 8%) during the lockdown in England and Wales. Especially death at home (+ 35%) and deaths at care homes and hospices (+ 32%) show the greatest excess, suggesting that the public did not seek help in acute cardiovascular emergencies or that excess cardiovascular mortality was a result of undiagnosed COVID-19 [8].

In addition to the general observation of a decrease in emergency consultations, trends of admissions with specific medical conditions have been published. Several studies reported that admission numbers decreased for cardiovascular emergencies such as acute coronary syndrome (ACS), both ST elevation myocardial infarction (STEMI) and non-ST elevation myocardial infarction (NSTEMI) [19–25], as well as for stroke and transient ischemic attack (TIA) [26–29]. Evidence about trends for diagnoses other than cardiovascular diseases is scarce. However, this information might be crucial to inform targeted measures to mitigate possible deleterious effects of delays in emergency care in the current or future periods of social distancing.

## Methods

### Study design and population

In this analysis, we aimed to assess differential trends of admission counts in A&E departments in Germany during the COVID-19 pandemic according to age group, sex, location of the admitting hospital, and underlying medical condition. We sought to compare admission rates between 12.03.2020 and 30.06.2020 to the same period in 2019. Routine data of all admissions in A&E departments between 02.12.2019–30.06.2020 and between 01.12.2018–30.06.2019 were collected from six hospitals in five cities throughout Germany. This secondary data analysis was performed in accordance with the 1964 Declaration of Helsinki and its later amendments. Informed consent was not required in accordance with German legislation.

We aimed to approximately represent all geographic regions. We included hospitals from north-western, eastern, south-western, and south-eastern Germany. Three hospitals were included in the south-eastern region. In 2012, the city of hospital A was in the 5th quintile of the German Index of Socioeconomic Deprivation (highest deprivation), the cities of hospitals B and E were in the 3rd quintile, the city of hospital F was in the 2nd quintile, and the cities of hospitals C and D were in the 1st quintile (lowest deprivation) [30]. Moreover, the administrative district surrounding hospital F was of the sparsely populated rural type according to the classification of the German Federal Institute for Research on Building, Urban Affairs and Spatial Development [31, 32]. The administrative districts surrounding hospitals A-D were rural districts with a tendency of increasing population density, and finally, hospital E was surrounded by an urban administrative district [31, 32].

In Germany, hospitals are classified into basic, standard and maximum care providers [33]. All hospitals in this study were standard or maximum care providers. No hospital was a university hospital. All admissions at A&E departments were included in this study. In Germany, patients can present to A&E following three different routes: self-decided presentation to an A&E department of choice, after advice of a physician, or by an ambulance. After admission to A&E, patients are triaged, examined and given a working diagnosis. Based on this diagnosis, a decision is made whether the patient needs to be admitted to a hospital ward or can safely be discharged to an outpatient setting. A patient is classified as “outpatient”, if treated within an A&E department and discharged without staying in a hospital ward overnight. In certain instances, patients may be admitted to an observation ward attached to A&E for an extended monitoring. Those patients are considered “inpatient” although having been treated in A&E only. Finally, patients who are scheduled for a non-urgent hospital-based treatment can either be admitted directly to a general ward or by bypassing the A&E department. During the first phase of the pandemic, there was a call to cancel any planned or non-urgent hospital-based treatment in Germany [2].

### Assessment of variables

All admissions of the periods 01.12.2018–30.06.2019 and 02.12.2019–30.06.2020 were retrieved from the administrative databases of the selected hospitals. The 02.12.2019 was selected as first day of the second period to yield an identical number of days in both periods since 2020 was a leap year. We chose to define the time period prior to the 12.03.2020 as time before the COVID-19 pandemic because German authorities communicated restrictions in the health sector in response to rising cases with COVID-19 on the 12.03.3020 [2]. Data on day of admission, age, sex, case status (inpatient/outpatient) and main diagnosis (diagnosis at discharge) according to ICD-10 were available for analysis. Day of admission, sex, and case status had no missing observations. Fourteen cases had a missing observation on age, and were excluded from this analysis. 8.1% of all cases had no information on main diagnosis according to ICD-10. We categorised age into the age groups 0–17, 18–64, and over 64 years.

For stratified analysis, we chose to investigate a pre-defined set of 10 diagnosis groups using ICD-10 codes with the aim to represent both a heterogeneous spectrum of common diseases in A&E departments and urgent emergencies. First, we selected acute myocardial infarction (AMI), stroke or TIA, and chronic obstructive pulmonary disease (COPD) since these diagnoses were investigated in a recent study of A&E admissions in Germany [7]. Mental and behavioural disorders due to use of alcohol (MDA) were included because this diagnosis was the most common psychiatric diagnosis in the included A&E departments, and because prevalence of mental disorders increased after implementation of the lockdown. Pneumonia was included because incidence of infectious respiratory diseases decreased in Germany after the introduction of social distancing. In addition, we chose to investigate a common injury that is mainly acquired at home (fractures of the femur) and an injury that is usually acquired outside the home (fractures of the forearm). Finally, heart failure, back pain, and cholelithiasis or cholecystits were included to represent the most common cardiovascular, orthopaedic, and gastrointestinal diagnoses in the included A&E departments. The defined diagnosis groups with corresponding ICD-10 codes were: MDA (ICD-10: F10), AMI (ICD-10: I21, I22), stroke or TIA (ICD-10: I61, I63, I64, G45), heart failure (ICD-10: I50), viral, bacterial, or unspecified pneumonia (ICD-10: J12-J18), COPD (ICD-10: J44), cholelithiasis or cholecystitis (ICD-10: K80, K81), back pain (ICD-10: M54), fracture of the forearm (ICD-10: S52), and fracture of the femur (ICD-10: S72).

### Statistical analyses

Admission counts at all emergency departments were plotted for periods of 2 weeks to show the development of admissions over time. To compare admission counts, we calculated rate ratios (RR) with 95% confidence intervals (95% CI) assuming no change in the underlying population between identical time periods in 2020 and 2019. 2019 was the year of reference. RR were adjusted for hospital, age group, and sex using Poisson regression. To assess a trend in admission rates between 2020 and 2019 independent from the COVID-19 outbreak, we compared the period 02.12.2019–12.03.2020 with 01.12.2018–12.03.2019. To assess differential trends, we calculated RR comparing the entire period between 12.03. and 30.06. of 2020 with 2019, stratified by age, sex, and hospitals. Concerning admissions according to diagnosis groups, we compared the first 56 days after 11.03. (12.03.-06.05.), and the remaining 55 days (07.05.-30.06.) between 2020 and 2019 in order to gain insight into the trajectories of admissions at A&E.

## Results

We registered 41,353 admissions at A&E departments in the period 12.03.2020 to 30.06.2020 (Table 1). During the same calendar time in 2019, 51,030 admissions were registered in the included hospitals. In addition, there was data on 46,114 admissions during the period preceding the 12.03. in 2020 and 45,701 in 2019. Among all included admissions, 4.2% were under the age of 18, 50.4% were male, and 50.0% were treated as outpatient cases. The data included approximately equal numbers of admissions from five hospitals, while there were lower numbers in hospital F (11.0%). Stroke or TIA, heart failure, and back pain were the most frequent observed diagnosis groups (Table 2).

**Table 1:** Descriptive characteristics of admitted cases in all included accident and emergency departments

	01.12.2018–11.03.2019	12.03.2019–30.06.2019	02.12.2019–11.03.2020	12.03.2020–30.06.2020	Overall
	N (%)	N (%)	N (%)	N (%)	N (%)
	45,701 (100.0%)	51,030 (100.0%)	46,114 (100.0%)	41,353 (100.0%)	184,198 (100.0%)
**Age group**					
0–17	1685 (3.7%)	2554 (5.0%)	1703 (3.7%)	1777 (4.3%)	7719 (4.2%)
18–64	24,885 (54.5%)	28,244 (55.3%)	24,779 (53.7%)	22,345 (54.0%)	100,253 (54.4%)
over 64	19,131 (41.9%)	20,232 (39.6%)	19,632 (42.6%)	17,231 (41.7%)	76,226 (41.4%)
**Male sex**	22,899 (50.1%)	26,005 (51.0%)	23,000 (49.9%)	20,893 (50.5%)	92,797 (50.4%)
**Hospital**					
A	7614 (16.7%)	8530 (16.7%)	7384 (16.0%)	7769 (18.8%)	31,297 (17.0%)
B	7491 (16.4%)	8152 (16.0%)	7784 (16.9%)	7414 (17.9%)	30,841 (16.7%)
C	9861 (21.6%)	10,948 (21.5%)	10,049 (21.8%)	8068 (19.5%)	38,926 (21.1%)
D	7835 (17.1%)	8744 (17.1%)	7829 (17.0%)	6533 (15.8%)	30,941 (16.8%)
E	8021 (17.6%)	8797 (17.2%)	8078 (17.5%)	7084 (17.1%)	31,980 (17.4%)
F	4879 (10.7%)	5859 (11.5%)	4990 (10.8%)	4485 (10.8%)	20,213 (11.0%)
**Outpatient case**	22,375 (49.0%)	26,233 (51.4%)	23,122 (50.1%)	20,403 (49.3%)	92,133 (50.0%)

**Table 2:** Diagnosis groups of admitted cases in all included accident and emergency departments

	01.12.2018–11.03.2019	12.03.2019–30.06.2019	02.12.2019–11.03.2020	12.03.2020–30.06.2020	Overall
	N (%)	N (%)	N (%)	N (%)	N (%)
	45,701 (100.0%)	51,030 (100.0%)	46,114 (100.0%)	41,353 (100.0%)	184,198 (100.0%)
**Diagnosis groups**					
Mental disorders due to use of alcohol (ICD-10: F10)	419 (0.9%)	444 (0.9%)	367 (0.8%)	255 (0.6%)	1485 (0.8%)
Acute myocardial infarction (ICD-10: I21, I22)	709 (1.6%)	652 (1.3%)	703 (1.5%)	643 (1.6%)	2707 (1.5%)
Stroke or TIA (ICD-10: I61, I63, I64, G45)	1028 (2.2%)	1209 (2.4%)	1091 (2.4%)	986 (2.4%)	4314 (2.3%)
Heart failure (ICD-10: I50)	978 (2.1%)	1054 (2.1%)	1050 (2.3%)	888 (2.1%)	3970 (2.2%)
Any pneumonia (ICD-10: J12-J18)	714 (1.6%)	676 (1.3%)	852 (1.8%)	914 (2.2%)	3156 (1.7%)
Chronic obstructive pulmonary disease (ICD-10: J44)	432 (0.9%)	418 (0.8%)	394 (0.9%)	292 (0.7%)	1536 (0.8%)
Cholelithiasis or cholecystitis (ICD-10: K80, K81)	443 (1.0%)	472 (0.9%)	466 (1.0%)	459 (1.1%)	1840 (1.0%)
Back pain (ICD-10: M54)	870 (1.9%)	1009 (2.0%)	899 (1.9%)	653 (1.6%)	3431 (1.9%)
Fracture of forearm (ICD-10: S52)	467 (1.0%)	593 (1.2%)	412 (0.9%)	535 (1.3%)	2007 (1.1%)
Fracture of femur (ICD-10: S72)	466 (1.0%)	477 (0.9%)	496 (1.1%)	477 (1.2%)	1916 (1.0%)
Missing ICD-10 code	3684 (8.1%)	4251 (8.3%)	3794 (8.2%)	3249 (7.9%)	14,978 (8.1%)

**Fig. 1:**
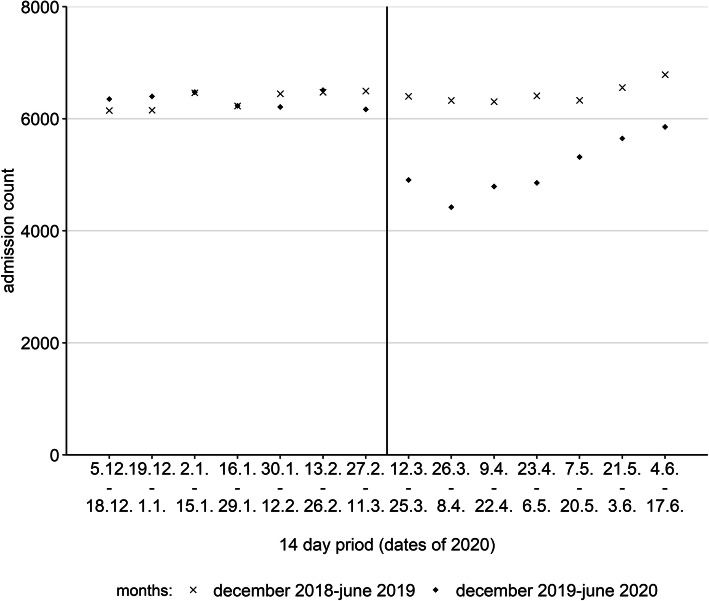
Overall admitted cases per periods of 2 weeks in 2019/2020 compared to 2018/2019

Biweekly numbers of admissions in 2020 and 2019 are displayed in Fig. 1. When comparing the entire time period prior to 12.03. between 2020 and 2019, there was no evidence of a difference (RR: 1.01, 95% CI: 1.00–1.02). We observed a RR of 0.95 (95% CI: 0.92–0.98) when comparing the 2 weeks prior to the 12.03. in 2020 with 2019. The RR was 0.77 (95% CI: 0.74–0.80) when comparing weeks 1 and 2 after 12.03., and 0.70 (95% CI: 0.67–0.73) when comparing weeks 3 and 4 after 12.03.between 2020 and 2019. The relative decrease in weeks 3 and 4 was the maximum decline in the observed period. After this point, admission counts converged, but did not reach levels of 2019 in June 2020. In the period 12.03.-30.06., the overall RR was 0.81 (95% CI: 0.80–0.82).

**Fig. 2:**
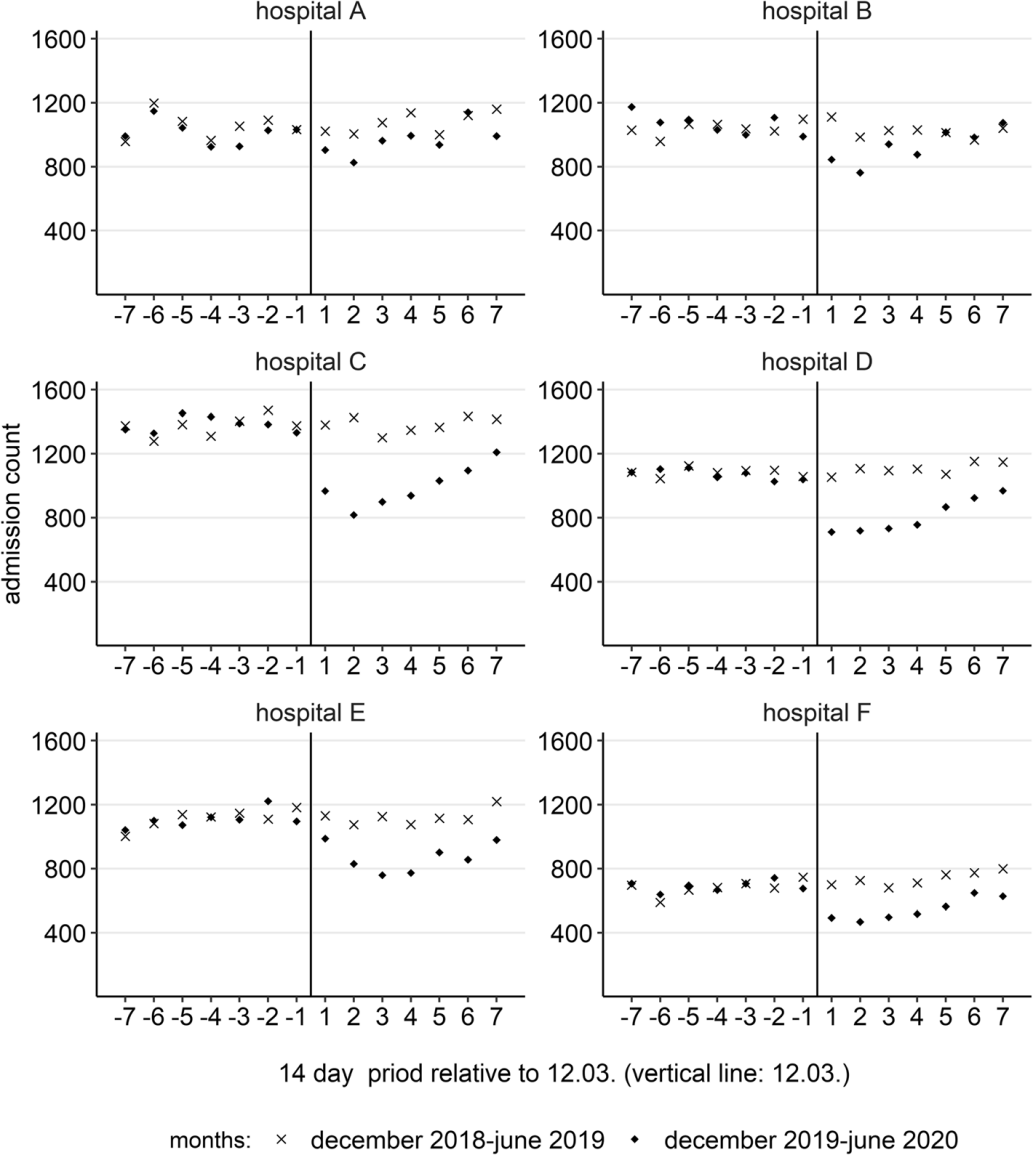
Admitted cases per periods of 2 weeks in 2019/2020 compared to 2018/2019 stratified by hospital. Legend of time periods: **“**-7”: 5.12.-18.12.; “-6”: 19.12.-1.1.; “-5”: 2.1.-15.1.; “-4”: 16.1.-29.1.; “-3”: 30.1.-12.2.; “-2”: 13.2.-26.2.; “-1”: 27.2.-11.3.; “1”: 12.3.-25.3.; “2”: 26.3.-8.4.; “3”: 9.4.-22.4.; “4”: 23.4.-6.5.; “5”: 7.5.-20.5.; “6”: 21.5.-3.6.; “7”: 4.6.-17.6

Concerning differential trends of admission counts when comparing the period 12.03.2020–30.06.2020 with the same period in 2019 according to sex, age, case status and hospital, we observed no meaningful differences for sex and case status. The RR among females was 0.82 (95% CI: 0.80–0.83), while the RR among males was 0.80 (95% CI: 0.79–0.82). Among inpatient cases, we observed a RR of 0.84 (95% CI: 0.83–0.86). Among outpatient cases, we observed a RR of 0.78 (95% CI: 0.76–0.79). Turning to age, we observed a larger relative decrease among the youngest age group of 0–17 year olds (RR: 0.70, 95% CI: 0.65–0.74) compared to older age groups. Among 18–64 year olds, the RR was 0.79 (95% CI: 0.77–0.81), and among people aged over 64 we observed a RR of 0.85 (95% CI: 0.83–0.87). Finally, we observed a more moderate decrease of admission counts in hospitals A, B, and E, compared to hospitals C, D, and F. The RR in hospital A was 0.91 (95% CI: 0.88–0.94), in hospital B 0.91 (95% CI: 0.88–0.94), and in hospital E 0.81 (95% CI: 0.78–0.83), in contrast to a RR of 0.74 (95% CI: 0.72–0.76) in hospital C, 0.75 (95% CI: 0.72–0.77) in hospital D, and 0.77 (95% CI: 0.74–0.80) in hospital F (Fig. 2).

When investigating differential trends of admissions according to diagnosis group, we found a heterogeneous picture. During the first 56 days after 12.03., there was a decrease of admissions with all diagnoses except pneumonia and fractures of the femur (Table 3). The admission rate of cases with pneumonia was higher compared to 2019 in this period. The decline of admissions with MDA and back pain was significantly larger compared to all admissions. During the 55-day period 07.05.-30.06., on the other hand, the decrease of admissions with MDA and back pain was still larger compared to all admissions. Admission rates with stroke or TIA and COPD were still lower compared to 2019. In addition, admissions with pneumonia were lower compared to 2019 in this later period. Admissions with heart failure, fractures of the forearm, and fractures of the femur were not different from counts in 2019. Finally, admissions with AMI and cholelithiasis or cholecystitis were higher than in 2019.

**Table 3:** Admissions in 2019/2020 compared to 2018/2019 during two periods according to diagnosis groups

12.03.-06.05. (period A)				07.05.-30.06. (period B)			
rate ratio	95% CI			rate ratio	95% CI		
All admissions							
0.75	0.73	to	0.76	0.87	0.86	to	0.89
Mental disorders due to use of alcohol (ICD-10: F10)							
0.47	0.37	to	0.59	0.67	0.55	to	0.82
Acute myocardial infarction (ICD-10: I21, I22)							
0.85	0.73	to	1.00	1.13	0.97	to	1.32
Stroke or TIA (ICD-10: I61, I63, I64, G45)							
0.76	0.67	to	0.86	0.87	0.77	to	0.97
Heart failure (ICD-10: I50)							
0.73	0.65	to	0.83	0.97	0.85	to	1.10
Viral, bacterial or unspecified pneumonia (ICD-10: J12-J18)							
1.85	1.63	to	2.11	0.77	0.65	to	0.91
Chronic obstructive pulmonary disease (ICD-10: J44)							
0.63	0.51	to	0.76	0.80	0.64	to	1.01
Cholelithiasis or cholecystitis (ICD-10: K80, K81)							
0.76	0.63	to	0.92	1.20	1.01	to	1.44
Back pain (ICD-10: M54)							
0.51	0.44	to	0.59	0.79	0.69	to	0.91
Fracture of forearm (ICD-10: S52)							
0.77	0.65	to	0.91	1.04	0.88	to	1.22
Fracture of femur (ICD-10: S72)							
1.08	0.91	to	1.29	0.92	0.76	to	1.10

Period A: 12.03.-06.05.2020 compared to 12.03.-06.05.2019.

Period B: 07.05.-30.06.2020 compared to 07.05.-30.06.2019.

Rate ratios were adjusted for hospital, sex and age group.

95% CI: 95% confidence interval.

## Discussion

In our analysis of differential trends of admission counts at A&E departments in Germany between 12.03.2020 and 30.06.2020 compared to the same period in 2019, we observed an average decline of admissions of 19%. The maximal decline of 30% occurred in weeks 3 and 4 after 12th March 2020. After that point in time, admission counts converged, but did not reach levels of 2019 in June 2020. There was some evidence that admission counts already declined in the 2 weeks prior to 12.03.2020. The relative decline was stronger for the age group 0–17 years and varied according to location of the hospital. Finally, in the first 8 weeks after 12.03., admission counts of all diagnoses except femur fractures and pneumonia declined. Between 07.05. and 30.6.2020, we found that rates for heart failure and fractures of the forearm converged to levels in 2019 and noted a moderate increase of counts of admissions with AMI and cholelithiasis or cholecystitis compared to the previous year.

Important limitations of our study are the imprecise case definition according to ICD-10 codes and a substantial proportion of cases with missing ICD-10 code. Validity of using ICD-10 codes to measure the acute medical condition leading to patients’ admission in A&E is rather low since classification of cases in German hospitals is done by accounting using defined algorithms. However, these algorithms are uniform across hospitals and have not changed between 2019 and 2020. Furthermore, they did not change during the outbreak of COVID-19. Hence, risk of differential misclassification is rather low and studies of trends are possible. The issue of missing observations for ICD-10 diagnoses can be interpreted analogously. Second, we compared trends among a large number of subgroups. Therefore, statistical precision of point estimates needs to be evaluated critically. In our study, precision of rate ratios was sufficient to examine differential trends of pre-selected subgroups. In some periods, we found that trends of admission rates of MDA, AMI, pneumonia, cholelithiasis or cholecystitis, back pain, and fractures of the femur differed from the average trend of all admissions since confidence intervals did not overlap. Moreover, the area of south-eastern Germany was overrepresented in our analysis. Since no included hospital was university hospital, our analysis complements a previous study from Germany that mainly included university hospitals [7].

Our results are in line with international studies that described a decline of admissions with stroke [26, 29], and TIA [28]. Furthermore, our result of a decline of admissions with stroke confirms a study from Germany that used health insurance claims data [27] and a national study using the identical ICD-10 codes for stroke and TIA as in this analysis [7]. Moreover, our observation of lower admissions with AMI during the first 8 weeks after 12.03. is in line with international [19–22, 24, 25] and national studies [7]. In international studies, a decline of admissions was observed for all types of acute ischemic heart diseases [24] and when using the ICD-10 codes I21 and I22 [26]. A previous study from Germany used the ICD-10 code I21, and found a decline of admissions with AMI using data until 30.05. across 36 A&E departments, 29 of which were university hospitals [7]. However, we found indications of an increase of admissions with AMI and cholelithiasis or cholecystitis between 07.05. and 30.06.

Our results only allow to state hypotheses about causes of the observed trends. The rise of admissions with AMI and cholelithiasis or cholecystitis in the late period of our study could be explained with the cancellation of some measures of social distancing. Among others, the federal states of Brandenburg, Bayern, and Berlin decided to reduce some measures of social distancing between 06.05. and 09.05 [34–36]. Furthermore, our findings suggest that delays of emergency care for patients with AMI and cholelithiasis or cholecystitis in the early phase of the pandemic could have led to a higher need for care later on. On the other hand, an increased incidence of these diseases would also explain our observation. Furthermore, we hypothesise that the larger decline of admissions in the age group 0–17 years compared to older age groups could be explained by a decrease of consultations with low urgency since it has been suggested that young patients in A&E in Germany report a low subjective therapeutic urgency more often than older patients [37]. Finally, the call to cancel any non-urgent hospital-based treatment might explain a share of the decrease of admissions at A&E [2]. Since admission counts of outpatients and inpatients declined to a similar degree in our study, the reorganisation of hospital-based treatment cannot explain the entire decline of admissions at A&E.

Concerning trends of admissions with injuries, we expected a decline of admissions with fractures of the forearm because incidence of traumatic injuries that usually occur outside the home probably declined during lockdown [10, 11]. This hypothesis was supported in our data, which showed a decline of admissions with fractures of the forearm in weeks 1 to 8 after 12.03., but unchanged admission rates of patients with fractures of the femur. In contrast to fractures of the forearm, femur fractures usually occur at home among elderly people. Moreover, the admission rate of patients with MDA was expected to rise since prevalence of mental disorders, psychological distress and alcohol use probably increased during the pandemic [12–16]. The strong decline of emergency admissions with MDA suggests that a considerable share of people with such disorders avoided attending A&E departments and might have received informal care at home. These observations support recent national and international calls for an increased attention to the burden of mental disorders during the COVID-19 pandemic [15, 38].

Finally, incidence of acute infections of the respiratory tract declined in Germany after the lockdown [9]. Considering this observation, aspects other than incidence probably contributed to the detected increase of admissions with pneumonia in the early period of our study. Soon after the onset of the pandemic, CT scans were introduced to assess patients with symptoms of acute respiratory tract infection because a more accurate diagnosis of COVID-19 was expected [39]. We hypothesise that this change of the diagnostic routine soon after the outbreak of COVID-19 may have led to an initial over-detection of pneumonia. The decline of admissions with pneumonia 9 to 16 weeks after 12.03., on the other hand, might partially be explained by the decline of incidence of respiratory tract infections. Lower incidence of respiratory tract infections might, furthermore, partially explain the decrease of admissions with COPD since infection-related exacerbations probably became less likely.

The observed regional variation of trends of admissions in A&E point towards an important role of context. Burden of incident infections with COVID-19 differed between administrative regions in Germany and might have influenced health-seeking behaviour differentially in each region. However, average incidence rates of COVID-19 during the peak of the pandemic in each area is not clearly related with the magnitude of the local decrease in admissions at A&E that we found in our study [40]. Hence, local burden of COVID-19 incidence did not explain the extent of the local decline in admissions at A&E departments. Similarly, rurality of the administrative districts surrounding each hospital was not related with local admission trends [31, 32]. Regional socioeconomic deprivation, on the other hand, might be a better explanation. The three cities with the largest relative decrease of admissions showed a lower degree of socioeconomic deprivation compared to the three hospitals with low relative decreases [30]. However, further regional characteristics that might have influenced admissions at A&E departments should be investigated more thoroughly in future research.

Finally, it is important to assess if delays in emergency care are accompanied with a rise of local mortality. Local mortality rates are available from statistical offices of the federal states [41–45]. All-cause mortality per 1000 population in 2019 was 12 in the catchment areas of hospitals A and E, 10 in hospitals B and F, and 9 in hospitals C and D. Monthly all-cause mortality from 2020 is available from the catchment areas of hospitals A, B, and F [41–45]. In the catchment area of hospital A, the mortality of the period March to June 2020 was 13 per 1000, compared to 12 per 1000 in the same period in 2019. Mortality rates of both periods in the catchment area of hospital B and F were identical in 2020 and 2019. These crude comparisons among three hospitals do not suggest an increase of local all-cause mortality. However, more detailed analyses and an assessment of long-term trends of all-cause and cause-specific mortality in areas with large decreases of emergency admissions might aid to gain insights about possible deleterious impacts of delayed emergency care.

## Conclusions

The observed decline of admissions with eight selected diagnoses after the initiation of the national lockdown in Germany is worrying since timely care might have been missed for some subgroups of these patients. We hypothesise that patients with MDA, AMI, stroke or TIA, heart failure, cholelithiasis or cholecystitis, and back pain avoided seeking emergency care. Declining incidence of respiratory tract infections and accidents outside the home might partially account for the decline of admissions with pneumonia, COPD, and fractures of the forearm. The increase of admissions with AMI and cholelithiasis or cholecystitis in weeks 9 to 16 after 12.03. indicates that possible delays of emergency care during the first 8 weeks of lockdown might have led to a rise of consultations for these diagnoses later on. In conclusion, we suggest to employ effective and targeted communication strategies that assert safety as well as the importance of immediate emergency care to the public during periods of social distancing.

## Data Availability

The clinical data of this study cannot be made publicly available because current data protection regulations in Germany prohibit publication of individual information, as anonymized information could still be used in combination and/or with other data to identify study participants. However, data can be made available for researchers who meet the criteria for access to confidential data upon reasonable request to the authors (e-mail: info.sozepi@mhb-fontane.de).

## Abbreviations

95% CI: 95% confidence interval
A&E: Accident and emergency
ACS: Acute coronary syndrome
AMI: Acute myocardial infarction
COPD: Chronic obstructive pulmonary disease
COVID-19: Coronavirus disease 2019
ICD-10: International Statistical Classification of Diseases and Related Health Problems, version 10
MDA: Mental and behavioural disorders due to use of alcohol
NSTEMI: Non ST-elevation myocardial infarction
RR: rate ratio
SARS-CoV-2: Severe acute respiratory syndrome coronavirus type 2
STEMI: ST-elevation myocardial infarction
TIA: Transient ischemic attack
